# Increased EPA levels in serum phospholipids of humans after four weeks daily ingestion of one portion chicken fed linseed and rapeseed oil

**DOI:** 10.1186/1476-511X-11-104

**Published:** 2012-08-22

**Authors:** Anna Haug, Nicole F Nyquist, Therese J Mosti, Malin Andersen, Arne T Høstmark

**Affiliations:** 1 Department of Animal and Aquacultural Sciences, The Norwegian University of Life Sciences, P.O.BOX 5003, Ås, 1432, Norway; 2 Section of Preventive Medicine and Epidemiology, University of Oslo, Blindern, P.O.BOX 113, Oslo, 0318, Norway

**Keywords:** n-3 fatty acids, Serum phospholipids, EPA, Arachidonic acid, Chicken meat, Feed, Linseed oil, Rapeseed oil, Soybean oil

## Abstract

Since the amounts of arachidonic acid (AA) and EPA in food may have implications for human health, we investigated whether a small change in chicken feed influenced the blood lipid concentration in humans ingesting the chicken. Forty-six young healthy volunteers (age 20–29) were randomly allocated into two groups in a double-blind dietary intervention trial, involving ingestion of about 160 g chicken meat per day for 4 weeks. The ingested meat was either from chickens given a feed concentrate resembling the commercial chicken feed, containing 4% soybean oil (SO), or the meat was from chickens given a feed where the soybean oil had been replaced by 2% rapeseed oil plus 2% linseed oil (RLO).

Serum total cholesterol, LDL and HDL cholesterol, triacylglycerols, serum phospholipid fatty acid concentration, blood pressure, body weight and C-reactive protein were determined at baseline and post-intervention. In subjects consuming chicken meat from the RLO group there was a significantly (p < 0.001) increased concentration of EPA in serum phospholipids, and a reduced ratio between AA and EPA. The participants that had a low% of EPA + DHA in serum phospholipids (less than 4.6%), all increased their% of EPA + DHA after the four week intervention period when consuming the RLO chicken. No significant response differences in cholesterol, triacylglycerol, C-reactive protein, body weight or blood pressure were observed between the groups. This trial demonstrates that a simple change in chicken feed can have beneficial effects on amount of EPA and the AA/EPA ratio in human serum phospholipids.

## Background

Chicken meat is popular to eat, and it is regarded as a healthy type of meat [1]. In Norway poultry meat accounts for about 25% of the total meat intake [2,3], and the consumption of this type of meat has shown an increasing trend. Meat is one of the food items that are good carriers of long chain polyunsaturated fatty acids (LC PUFAs). The fatty acid composition of chicken meat is affected by the type of fat in the chicken feed. Commercial chicken feed is based on grains and soybean oil rich in n-6 fatty acids and the ratio of n-6 to n-3 in the feed is about 10–15/1. The potential of chickens to convert the n-6 and n-3 feed fatty acids to the respective long chain n-6 and n-3 fatty acids has been studied intensively, showing that the concentration of n-6 linoleic acid, (LA) and n-3 *alpha*-linolenic acid, (ALA) fatty acids in the feed influence the production of the long n-6 and n-3 fatty acids, such as arachidonic acid (AA), eicosapentaenoic acid (EPA), docosapentaenoic acid (DPA) and docosahexaenoic acid (DHA) in the chicken [4,5].

The n-6 and n-3 fatty acids compete for binding to enzymes, receptors and membranes affecting cell metabolism. Overproduction or imbalance (*e.g.* between thromboxanes and prostacyclins) of the different eicosanoids are implicated in the pathogenesis, symptom severity or rate of progression of several common chronic diseases, such as allergy, cardiac diseases, cancer, pain, rheumatoid arthritis, other inflammation and high blood pressure [6-12]. DHA has, moreover, an important structural role as a major component in membrane lipids in the brain, retina, testicles and spermatozoa. The reasons for this are not well understood, but it has been suggested that it is mainly for improving membrane fluidity in the mitochondria, thus facilitating electron transport through the respiratory chain and reducing the ratio between rates of mitochondrial production of reactive oxygen species (ROS) and ATP [7]. Even though much of the underlying biochemical mechanisms explaining the causal connection between dietary intakes of LC PUFAs, the dietary n-6/n-3 ratio and eicosanoid biosynthesis and disease processes have been known for more than 40 years, neither the agricultural sector nor preventive medicine seem to have shown much interest in making practical use of the information.

From known physiology, a combination of AA overconsumption and underconsumption of EPA and DHA should lead to enhancement of the death risk both from cardiovascular disease and cancer. Most of the red meat produced in the United States has a high n-6/n-3 ratio, *e.g.* pork leg (no 10010[13]) has about 25:1, beef (no 23652[13]) has 18:1, and chicken meat (no 05011[13]) has 8:1. However, grass fed beef (no 13000[13]) is shown to have an n-6/n-3 ratio of only 2:1. The same low ratio is shown in sheep meat from *e.g.* Iceland, where grass feeding is commonly practiced, being about 1.8:1[14]. The association between red meat consumption and death risk from cardiovascular disease or cancer in populations who consume large quantities of red meat with a natural fatty acid composition, has not yet been well enough systematically evaluated.

When comparing the intakes of n-6 and n-3 fatty acids in the western diet with the intake in earlier times, it seems to be a higher concentration of n-6 and lower n-3 fatty acid concentration in meat, offal and eggs today (because the n-6/n-3 ratio of the animal diet is now unnaturally high) [6]. The high n-6 content in feeds leads to increased synthesis of AA in the animals, and an increased load of AA for the consumer. A change in the n-6 and n-3 balance in the animal feed back to more natural concentrations is healthier for the animals, and it is safe. It is also without side effects (which all commonly used pharmacological inhibitors of eicosanoid synthesis do have) and cheaper for the consumers than to take drugs for dampening eicosanoid synthesis. It is technologically easy and relatively inexpensive to produce poultry meat with much more long-chain n-3 fatty acids and less arachidonic acid than now [15]. The health economic benefits of such livestock products for society as a whole may easily outweigh the direct costs for the poultry farming sector. It may be concluded that time is overdue for a better balanced intake of n-6 and n-3 fatty acids in food.

Practical ways to improve the fatty acid composition of chicken meat to contain less n-6 and more n-3 may be by excluding soybean oil from the commercial feed, and add rapeseed- and linseed oil instead. The n-6/n-3 ratio in the feed will thereby be reduced considerably, and the ratio in the chicken meat will improve. It has been shown that the ratio between the n-6 fatty acid AA and the n-3 fatty acid EPA in chicken breast muscle was about 3:1 when adding 4% rapeseed oil and 1% linseed oil to the feed [16].

The objective of the present study was to examine the effects of consuming a daily portion of chicken meat for four weeks; either meat from chickens fed a traditional feed supplemented with soybean oil, or feed with linseed- plus rapeseed oil, on the fatty acid composition and the ratio n-6/n-3 and AA/EPA in serum phospholipids, blood lipids, C-reactive protein and blood pressure in healthy 20–29 year old subjects.

## Methods

### Study design for human experiment

Forty-six healthy volunteers, 11 males and 35 females, aged 20 to 29 years were participating in the study. The study lasted for 28 days and was carried out in October-November 2011. Most of the participants were students at the Norwegian University for Life Science. The majority had normal BMI; individual BMI ranged from 17.5 to 33.5. They were not taking any medications, supplements or fish oil, and not eating fatty fish. The study was double blind and randomized. The participants were randomly allocated into one of two groups: to consume chicken meat from chickens that had been fed a concentrate feed supplemented with soybean oil or with rapeseed plus linseed oil. Some of the participants were living together in student flats, and they were allocated into the same group so they could cook together and eat the same type of chicken. The participants were recruited by two Masters students. The Masters students were not participating in the study themselves, and did not know the type of chicken feed. The participants were asked to continue with their normal dietary habits, and not to be slimming during the experimental period. Every week, two frozen chickens (the weight of a chicken was about 1 kg) were given to each participant. The participants were given recipes indicating how to cook the chickens. They told later in the study that they were not aware of how easy it was to cook the whole chickens.

### Approval of the study

This study was conducted according to the guidelines laid down in the Declaration of Helsinki and all procedures involving human subjects were approved by the Regional Committee for Medical Research Ethics and approved by the Norwegian Data Inspectorate. Written informed consent was obtained from all the subjects, and they were informed that they could quit the study whenever they wanted without giving any reason.

### Blood samples, anthropometric and blood pressure measurements

At baseline (day 1) and at post-intervention (day 28) the participants were weighed using the same scale (Soehnle Digital personal scale, 467017201, Germany) and blood pressure was measured using an automatic blood pressure monitor approved for medical purposes (UA-767 Plus 30. Blood pressure monitor, A&D, Japan). The blood pressure was taken three times according to the instruction manual. The height was measured at baseline using a wall mounted stadiometer, for calculation of BMI; weight, kg / (height, m)^2^.

Blood samples were collected from fasting subjects (minimum 12 h fast) at baseline and post-intervention (between 07.00 and 10.30). The blood samples were left for 0.5 – 2 h at room temperature before centrifuging at 1300 g for 12 minutes. Serum was then frozen and kept at -20°C until analyzed. Blood sampling and measuring of weight, height and blood pressure were done at the local medical health center.

### Serum analyses

Human serum phospholipid fatty acids were determined using the following method; serum was thawed overnight at 4°C and vortexed for 5 sec. Dichloromethane/methanol were added to 200μl serum and 100μl internal standard (1,2 diheptadecaonyl-*sn*-glycero-3-phosphatidylcholine). After shaking and centrifugation the supernatants were transferred to new vials and washed in 0.9% NaCl solution. Lower phases were transferred to SPE columns. Neutral lipids were washed out with dichloromethane /isopropanol and MTBE/formic acid. Phospholipids were eluted with methanol. After evaporation to dryness in a vacuum centrifuge, phospholipids were transmethylated with sodium metoxide and FAMEs were extracted to hexane before GC analysis. Analysis was performed on a 7890A GC with a split/split less injector, a 7683B automatic liquid sampler, and flame ionization detection (Agilent Technologies, Palo Alto, CA). Separation was performed on a SP 2380 (30 m × 0.22 mm i.d. × 0.25 μm film thickness) column (Supelco, Inc., Bellefonte, PA).

Determination of serum total cholesterol, HDL cholesterol (HDL), LDL cholesterol (LDL), triacylglycerol (TAG) and CRP was performed using routine laboratory methods (SP 03-05 Avida 2400. Fürst Medical Laboratory, Norway).

### The chicken feeding experiment; feed production and chickens

The animals were cared for according to laws and regulations controlling experiments with live animals in Norway (The Animal Protection Act of December 20^th^, 1974 and the Animal Protection Ordinance Concerning Experiments with Animals of January 15^th^, 1996).

The two types of chicken feed were produced at the Center for Feed Technology, Ås, Norway. The feed was based on wheat, and the two feed types; SO and RLO, were identical with the exception of supplemented oil, being either 4% soybean oil or 2% rapeseed oil (Askim bær- og fruktpresseri, Askim, Norway) plus 2% linseed oil (Naturata AG, Murr, Germany) (Table 1). The wheat was ground on a hammer mill, 3 mm sieve and the feed was pelleted with cold pelletation 3 mm, 600 kg/hour. The feed was packed in 500 kg sacks, and it was produced the same week as the onset of the feeding of the chickens. A small part of the feeds was ground, and this meal was given to the small chickens the first week. After the first week, the chickens were eating feed pellets.

**Table 1:** Chicken feed composition,% of ingredients

	**SO**	**RLO**
Wheat	45	45
Maize gluten	10	10
Soybean meal	17	17
Oat	15	15
Rendered fat	4	4
Soybean oil	4	0
Rape seed oil	0	2
Linseed oil	0	2
Choline cholride	0.13	0.13
Mono calcium phosphate	1.4	1.4
Ground limestone	1.3	1.3
Sodium chloride	0.25	0.25
Sodium bicarbonate	0.2	0.2
Mikromin Fjørfe FK50	0.15	0.15
Mikromin Selen 300FK	0.1	0.1
Vitamin A	0.03	0.03
Vitamin E	0.06	0.06
Vitamin ADBK	0.09	0.09
Vitamin D3	0.08	0.08
L-lysine	0.4	0.4
DL-methionine	0.2	0.2
L-threonine	0.2	0.2

The two diets were identical except the content of 4% soybean oil (SO) or 2% rapeseed oil plus 2% linseed oil (RLO).

Composition of Mikromin Fjørfe FK 50 is: 35 g Fe/kg, 10 g Cu/kg, 85 g Mn/kg, 55 g Zn/kg and 0.7 I /kg. Mikromin Selen 300 FK is: 0.3 g Se/kg. Both mixtures are from Normin AS, Hønefoss, Norway.

Six hundred newly hatched male broiler chickens (Ross 308, Nortura Samvirkekylling, Norway) were randomly divided into two rooms for chicken production. The floor was covered with wood shavings, and the chickens had free access to water and feed. The temperature in the two rooms was kept at 32°C for the first three days, before being reduced by 0.5°C per day until 21°C and then kept at this temperature until slaughter at day 32. During the initial 24 h the chickens were kept in continuous lighting, followed by six days with 23 h light and one h darkness. From day seven the lights were turned off for two periods per day; from 1700 to 2100 h, and 0000 to 0400 h. The chickens were inspected by a veterinarian each week. In total 13 birds died during the experiment; eight in the SO dietary treatment group, and five in the RLO dietary treatment group. This gives a total mortality rate of about 2%, which is acceptable. The dead chickens did not undergo post mortem autopsy.

At day 32, the chickens were sent to a commercial abattoir; Nortura kyllingslakteri, Rakkestad, Norway, and were slaughtered according to routine practice. The carcasses were frozen at -20°C. Three days later the human dietary intervention study started.

### Chicken feed and chicken breast muscle analyses

The fatty acid composition of chicken feed (six parallel samples from each feed) and chicken breast muscle (16 randomly chosen chickens from each group) was determined by extraction, methylation and gas chromatography according to O’Fallon et al. [17]. The fatty acid methyl esters (FAME) were separated on a fused silicium dioxide capillary column (200 m × 0.25 mm id). The carrier gas was H_2_, and the temperature program went from 70°C to 230°C. The detector temperature was 290°C, and the run time for each sample was 90 minutes. Fatty acids were identified with reference standard fatty acids (Sigma Aldrich, UK), and they were quantified by use of internal standard C13:0 (Sigma Aldrich, UK), that was added in the fatty acid extraction procedure.

### Statistical analyses

The fatty acid composition and fat content of feed and chicken muscle fatty acid were calculated using Excel. Mean value ± standard errors of the mean are presented. Independent Student’s t-test was used to compare baseline values of the two groups of human participants, and paired Student’s t-test to assess significance of the treatment effect in each group, for changes in serum cholesterol, triacylglycerols, HDL cholesterol, LDL cholesterol, C-reactive protein and fatty acid composition of serum phospholipids. Control for variation in baseline value was performed using ANCOVA. Bonferroni correction was used for multiple comparisons. We used the Statistical Analysis System, SAS 9.1, and results are presented as means ± SEM (or standard deviation when presenting the baseline characteristics). Differences were considered significant at p < 0.05.

## Results

### Participants

All of the 46 participants that were recruited for the study successfully completed the study. They consumed about 160 g chicken meat (raw weight) per day for four weeks, and they all gave a blood sample, measured blood pressure and weight at the beginning and at the end of the study.

### Basal characteristics

The number of men and women, age, mean and standard deviation of height, body weight, BMI, SBP, DBP, serum cholesterol, LDL cholesterol, HDL cholesterol, triacylglycerol and C-reactive protein of the participants in the SO group and RLO group at baseline is shown in Table 2. There were no significant differences among the two groups in basal characteristics, except the number of men being lower in the SO group compared to the RLO group. Out of the 46 participants in the study, there were only 11 men. Due to the situation that some of the participants were living together in flats, and the principle of having the same type of chicken in each household to ease the cooking, there were only three men in the group eating the soybean oil supplemented chicken, and eight men in the group having the rapeseed and linseed oil fed chicken.

**Table 2:** Basal characteristic of the study participants in SO and RLO groups (n = 23 in each group)

**Characteristics**	**SO**		**RLO**		P*
	**Mean**	**SD**	**Mean**	**SD**	
Sex					
Female (n)	20	-	15	-	-
Male (n)	3	-	8	-	-
Age (range) years	24 (19-29)	-	24 (19-29)	-	-
Height (m)	1.72	0.08	1.72	0.09	0.872
Weight (kg)	68.4	10.3	70.9	12.1	0.453
BMI (kg/m^2^)	23.1	2.7	23.9	3.7	0.419
SBP (mmHg)	117	13	120	13	0.399
DBP (mmHg)	74	11	73	10	0.503
Cholesterol (mmol/l)	4.7	1.0	4.9	0,6	0.529
LDL (mmol/l)	3.0	0.8	3.1	0.7	0.407
HDL (mmol/l)	1.7	0.3	1.7	0.4	0.966
TAG (mmol/l)	1.1	0.4	1.1	0.4	0.739
CRP (mg/l)	2.0	3.0	2.1	3.3	0.944

Body mass index (BMI), Systolic blood pressure (SBP), diastolig blood pressure (DBP), LDL cholesterol (LDL), HDL cholesterol (HDL) and C-reactive protein (CRP).

SBP = Systolic blood pressure.

DBP = Diastolic blood pressure.

*Significance in mean values between groups at baseline in one-way ANOVA.

### Chicken intake

The weight of the chickens was on average 1.09 kg. The participants individually made a diary describing their intake of chicken meat. In average both groups were eating 7.5 chickens during the four weeks of intervention, and they were having chicken most of the weekdays. A rough estimate made by us showed that under the given circumstances with simple kitchen facilities about 55% of the weight of raw chicken was consumed. Thus, the participants were consuming about 160 g chicken meat consisting in average of about 95 g breast muscle and 65 g thigh muscle per day. This corresponds to a moderate portion of chicken meat, a portion is estimated to be 175 g (raw meat) [18]. It has previously been shown that exchanging SO with RLO did not affect the taste and sensory aspects of the chicken meat (results not yet published).

### Fatty acid content in the chicken feed and meat

The composition of the feed given to the two chicken groups is show in Table 1, and the fatty acid composition of the feed is shown in Table 3. The feed composition was identical in the two diets with exception of the source of oils added to the diets; 4% soybean oil (SO) or 2% rapeseed oil plus 2% linseed oil (RLO). The fat content of the feed was 10.3%.

**Table 3:** Fatty acid composition of chicken feed,% fatty acid methyl ester (FAME)

	**SO**	**RLO**
C14:0	0.87	0.89
C15:0	0.17	0.18
C16:0	17.3	15.2
C16:1,n-7	1.10	1.16
C18:0	7.79	8.34
C18:1,c9	27.2	33.6
C18:2,n-6 (LA)	35.9	21.6
C18:3,n-3 (ALA)	3.61	12.33
C20:4,n-6 (AA)	0.06	0.09
C20:5,n-3 (EPA)	nd	nd
C22:5,n-3 (DPA)	0.04	0.04
C22:6,n-3 (DHA)	0.02	0.02
SFA	25.9	24.4
MUFA	28.3	34.8
PUFA	39.6	34.1
n-3 PUFA	3.67	12.39
n6/n3	9.82	1.75
16:1 n-7/16:0	0.06	0.08
18:1 c9/18:0	3.49	4.03

The diets contained 4% soybean oil (SO) or 2% rapeseed oil plus 2% linseed oil (RLO). (Means from six analyses of each feed).

The mean fatty acid content (mg fatty acid/100 g) chicken breast muscle of 16 chickens in each group is shown in Table 4. The mean fat content was 1.5 g fat/100 g breast muscle in both groups. The content of the fatty acids LA, ALA, AA, EPA, DPA and DHA was significantly different in the two groups of chicken breast fillets. The content of the other fatty acids in chicken breast meat was not different between the two groups. The sum of n-3 PUFA (ALA + EPA + DPA + DHA) and the sum of LC n-3 PUFA (EPA + DPA + DHA) was much higher in the meat from the chickens fed rapeseed plus linseed oil compared to the soybean oil group.

**Table 4:** Fatty acid concentration, mg/100 g of chicken breast filet from 16 animals fed a diet containing 4% soybean oil (SO) or 16 animals fed a diet containing 2% rapeseed oil plus 2% linseed oil (RLO)

	**SO**		**RLO**		P*
	**Mean**	**SEM**	**Mean**	**SEM**	
C14:0	9.75	1.20	9.85	1.03	0.951
C15:0	2.32	0.25	2.44	0.22	0.718
C16:0	307	28	287	25	0.585
C16:1 n-7	43.9	6.42	42.2	4.70	0.832
C18:0	142	10.31	137	10.44	0.736
C18:1,c9	451	55.45	507	53.6	0.469
C18:2,n-6 (LA)	379	42.78	245	21.0	0.009
C18:3,n-3 (ALA)	29.9	4.17	103	11.54	0.000
C20:4,n-6 (AA)	73.2	2.22	42.73	1.36	0.000
C20:5,n-3 (EPA)	4.54	0.24	21.19	0.61	0.000
C22:5,n-3 (DPA)	20.0	1.03	39.25	1.87	0.000
C22:6,n-3 (DHA)	14.1	0.97	20.41	1.10	0.000
SFA	459	39.74	433	36.0	0.634
MUFA	495	61.68	550	58.2	0.523
PUFA	521	48.04	472	34.8	0.420
n-3 PUFA	68.5	4.73	184	13.3	0.000
LC n-3 PUFA	38.6	1.72	80.9	2.97	0.000
AA/EPA	16.55	0.71	2.03	0.07	0.000
n-6/n-3	6.48	0.26	1.56	0.02	0.000
16:1n-7/16:0	0.13	0.01	0.14	0.00	0.240
18:1c9/18:0	3.03	0.18	3.65	0.13	0.010
AA/LA	0.23	0.03	0.19	0.01	0.146
EPA/ALA	0.22	0.05	0.23	0.02	0.879
DPA/EPA	4.52	0.28	1.86	0.09	0.000
DHA/DPA	0.72	0.05	0.53	0.02	0.001

(Mean values and standard errors, SEM).

*Significance in mean values between groups.

SFA: sum of 14:0, 16:0 and 18:0.

MUFA: sum of 16:1 n-7 and 18:1 n-9.

PUFA: sum of LA, ALA, AA, EPA, DPA and DHA.

n-3 PUFA: sum of ALA, EPA, DPA and DHA.

LC n-3 PUFA: sum of EPA, DPA and DHA.

n-6/n-3: (LA + AA)/(ALA + EPA + DPA + DHA).

The content of some minor and identified fatty acids; 17:0, 18:1 t6-11, 18:1c11, 20:0, 22:0, 18:3 n-6, 20:1 n-9, 20:2 n-6 and 20:3 n-6 are not shown in the Tables 3, 4, 5 and 6.

**Table 5:** Fatty acid composition of serum phospholipids at baseline and post-intervention in persons eating chickens fed soybean oil supplement (SO) and rapeseed and linseed oil supplement (RLO), (% of total fatty acids, FAME)

**Fatty acid**	**SO group (n = 23)**				**RLO group (n = 23)**				***P*§**	***P*¶**
	**Baseline**		**Post-intervention**		**Baseline**		**Post-intervention**			
	**Mean**	**SEM**	**Mean**	**SEM**	**Mean**	**SEM**	**Mean**	**SEM**		
C14:0	0.37	0.02	0.34	0.02	0.37	0.02	0.41	0.03	0.894	0.029
C15:0	0.23	0.01	0.21	0.01	0.25	0.01	0.25	0.01	0.127	0.004
C16:0	29.5	0.45	29.2	0.41	29.4	0.39	28.8	0.41	0.903	0.460
C16:1,n-7	0.67	0.05	0.61	0.04	0.57	0.04	0.57	0.03	0.114	0.455
C18:0	13.5	0.35	13.2	0.31	13.4	0.28	13.1	0.29	0.966	0.873
C18:1,c9	10.18	0.31	9.42	0.31	9.32	0.26	9.48	0.27	0.042	0.887
C18:2,n-6 (LA)	20.2	0.58	20.3	0.47	21.0	0.53	20.7	0.52	0.285	0.537
C18:3,n-3 (ALA)	0.27	0.02	0.23	0.01	0.26	0.02	0.36	0.03	0.735	0.000
C20:4,n-6 (AA)	8.41	0.39	10.21	0.46	9.16	0.36	9.66	0.34	0.167	0.340
C20:5,n-3 (EPA)	1.03	0.09	0.80	0.05	1.09	0.09	1.26	0.09	0.669	0.000
C22:5,n-3 (DPA)	0.91	0.05	0.92	0.05	0.92	0.05	1.08	0.06	0.836	0.065
C22:6,n-3 (DHA)	4.94	0.28	4.77	0.26	4.63	0.26	4.57	0.20	0.421	0.545
SFA	43.3	0.19	42.7	0.22	43.3	0.17	42.3	0.21	0.736	0.163
MUFA	10.8	0.32	10.0	0.32	9.89	0.29	10.0	0.29	0.034	0.967
PUFA	35.7	0.42	37.2	0.51	37.1	0.53	37.6	0.46	0.052	0.540
n-3 PUFA	7.15	0.35	6.72	0.29	6.90	0.34	7.26	0.27	0.614	0.187
LCn-3 PUFA	6.88	0.36	6.49	0.30	6.64	0.35	6.90	0.27	0.633	0.307
AA/EPA	9.25	0.74	13.84	1.07	9.73	0.89	8.72	0.88	0.679	0.001
n-6/n-3	4.23	0.23	4.73	0.22	4.62	0.25	4.33	0.19	0.251	0.166
16:1 n-7/16:0	0.02	0.00	0.02	0.00	0.02	0.00	0.02	0.00	0.100	0.542
18:1 c9/18:0	0.76	0.03	0.72	0.03	0.70	0.03	0.73	0.03	0.113	0.849
EPA/ALA	4.19	0.46	3.66	0.27	4.75	0.67	3.85	0.35	0.498	0.672
DPA/EPA	0.95	0.05	1.22	0.08	0.91	0.05	0.88	0.03	0.607	0.000
DHA/DPA	5.73	0.38	5.49	0.36	5.30	0.37	4.56	0.33	0.429	0.065

§Significance in mean values between groups at baseline.

¶ Significance in mean values between groups after the 4 week intervention period.

SFA: sum of 14:0, 16:0 and 18:0.

MUFA: sum of 16:1 n-7 and 18:1 n-9.

PUFA: sum of LA, ALA, AA, EPA, DPA and DHA.

n-3 PUFA: sum of ALA, EPA, DPA and DHA.

LC n-3 PUFA: sum of EPA, DPA and DHA.

n-6/n-3: (LA + AA)/(ALA + EPA + DPA + DHA).

**Table 6:** Fatty acid concentration (mg/100 ml serum) of serum phospholipids at baseline and post-intervention in persons eating chickens fed soybean oil supplement (SO) and rapeseed and linseed oil supplement (RLO)

	**SO group (n = 23)**				**RLO group (n = 23)**				***P*§**	***P*¶**
	**Baseline**		**Post-intervention**		**Baseline**		**Post-intervention**			
	**Mean**	**SEM**	**Mean**	**SEM**	**Mean**	**SEM**	**Mean**	**SEM**		
C14:0	0.42	0.03	0.39	0.03	0.43	0.04	0.46	0.03	0.889	0.158
C15:0	0.26	0.01	0.24	0.01	0.28	0.01	0.28	0.01	0.192	0.033
C16:0	33.99	1.47	33.49	1.60	33.73	1.23	31.87	1.20	0.889	0.423
C16:1,n-7	0.79	0.08	0.72	0.07	0.67	0.06	0.65	0.06	0.224	0.449
C18:0	15.34	0.58	15.00	0.62	15.30	0.50	14.53	0.56	0.958	0.576
C18:1,c9	11.64	0.50	10.82	0.63	10.75	0.55	10.56	0.54	0.238	0.757
C18:2,n-6 (LA)	22.90	0.76	23.00	0.86	23.96	0.88	22.89	0.91	0.368	0.932
C18:3,n-3 (ALA)	0.31	0.03	0.27	0.02	0.31	0.03	0.40	0.03	0.912	0.001
C20:4,n-6 (AA)	9.72	0.61	11.59	0.65	10.40	0.48	10.63	0.45	0.386	0.234
C20:5,n-3 (EPA)	1.20	0.13	0.92	0.08	1.23	0.10	1.40	0.10	0.860	0.001
C22:5,n-3 (DPA)	1.04	0.07	1.04	0.06	1.04	0.06	1.18	0.07	0.944	0.128
C22:6,n-3 (DHA)	5.74	0.44	5.44	0.38	5.26	0.31	5.04	0.26	0.382	0.395

§Significance in mean values between groups at baseline.

¶ Significance in mean values between groups after the 4 week intervention period.

When calculating ratios between the fatty acids in the chicken breast muscle, both AA/EPA and n-6/n-3 were significantly lower; about 8 times and 4 times lower, respectively, in the RLO meat compared to SO (Table 4).

### Fatty acid composition of human serum phospholipids

As shown in Table 5, there was no significant difference at baseline in mean *percentage* values of fatty acids (% FAME) in the serum phospholipids from persons in the SO and RLO group. In contrast to this, after intervention, ALA and EPA, as well as 14:0 and 15:0 were significantly higher in subjects eating the RLO chicken as compared with those eating the SO fed chicken. Additionally, the RLO group tended to have higher levels of DPA (p = 0.065). The post-intervention AA/EPA and DPA/EPA ratio was also significantly lower in the RLO group. Otherwise there were no significant differences between the two groups.

*Absolute* values of serum phospholipid fatty acids (-mg/100 ml serum) are shown in Table 6. The total amount of fatty acids is the same at baseline and post-intervention; about 110 mg phospholipid fatty acids/100 ml serum. There were no differences in fatty acid concentrations among the two groups at baseline. After intervention, the concentration of ALA, EPA and 15:0 was higher in serum from subjects eating RLO chicken as compared to subjects eating the SO chicken.

### Initial and post intervention values of body weight, blood pressure and serum variables

Mean body weight, BMI, SBP, DBP, serum cholesterol, LDL cholesterol, HDL cholesterol, triacylglycerol and C-reactive protein (CRP) of the participants at baseline and at the end of the intervention period (day 28) are shown in Table 7. There were no significant group differences in initial and post intervention values of body weight, BMI SBP, DBP, serum cholesterol, LDL cholesterol, HDL cholesterol, triacylglycerol and CRP, at the start of the study compared to end of the intervention period.

**Table 7:** Weight, BMI, systolic blood pressure (SBP), diastolic blood pressure (DBP), total cholesterol, LDL cholesterol (LDL), HDL cholesterol (HDL), triacylglycerol (TAG) and C-Reactive Protein (CRP) of study participants at baseline and post-intervention in SO and RLO treatment groups (n = 23 participants in each group)

	**SO group (n = 23)**				**RLO group (n = 23)**				***P*§**	***P*¶**
	**Baseline**		**Post-intervention**		**Baseline**		**Post-intervention**			
	**Mean**	**SEM**	**Mean**	**SEM**	**Mean**	**SEM**	**Mean**	**SEM**		
Weight (kg)	68.39	2.15	68.57	2.15	70.90	2.53	71.31	2.52	0.453	0.414
BMI (kg/m^2^)	23.11	0.57	23.17	0.57	23.89	0.77	24.02	0.77	0.419	0.376
SBP (mmHg)	116.7	2.30	115.5	1.93	119.9	2.42	117.9	2.15	0.399	0.366
DBP (mmHg)	73.63	1.60	70.7	1.40	73.2	1.84	71.3	1.84	0.503	0.903
Cholesterol (mmol/l)	4.72	0.20	4.59	0.18	4.87	0.13	4.68	0.13	0.529	0.681
LDL (mmol/l)	2.95	0.17	2.88	0.16	3.13	0.15	3.05	0.12	0.407	0.408
HDL (mmol/l)	1.65	0.06	1.61	0.07	1.66	0.08	1.60	0.08	0.966	0.902
TAG (mmol/l)	1.10	0.08	1.10	0.13	1.06	0.09	1.06	0.10	0.739	0.810
CRP (mg/l)	1.99	0.62	2.53	0.68	2.06	0.69	2.38	0.64	0.944	0.872

§Significance in mean values between groups at baseline.

¶ Significance in mean values between groups after the 4 week intervention period.

The body weight of participants increased in average about 0.3 kg during the intervention period, but this change was not statistically significant.

Serum cholesterol varied from 3.0 mmol/l (in one of the males), to 7.6 mmol/l (in one of the females). The CRP was below 10 mg/l in all except three readings, and two of the values were just above 10. One participant had CRP of 173 mg/l. This reading was removed from the data. All the other analyzed values of these persons were not extremes, and we chose not to remove any other values. The participants with values outside the given reference for their age group were informed about the findings and advised to see their personal physician for a check.

## Discussion

The present study shows that a daily intake of a moderate portion of chicken meat for 4 weeks can appreciably increase the concentration of EPA in serum phospholipids of young healthy humans, provided that the chickens had been fed rapeseed and linseed oil instead of similar amounts of soybean oil.

The daily intake of chicken meat, about 160 g/day, is much higher than the average daily intake of chicken meat in the Norwegian population; being about 50 g/day [2,3]. The total average intake of meat per person in Norway is estimated in two different reports to be about 130 g/day [2], and 200 g /day [3]. The subjects were advised to follow their normal diet, but to eat as much as possible of the two chickens they received each week, in preference to other meats. Their average total meat intake may therefore have been somewhat higher than 160 g/day during the study. In the present study the participants were mostly students at The Norwegian University of Life Sciences, living in student accommodation houses and having a limited budget. Since meat is expensive compared to cereal based food, they are likely to have less meat in their regular diet than the average Norwegian intake. Some of the students at The Norwegian University of Life Sciences are taking a course in nutrition where they undergo a dietary assessment showing that they have a diet based on much bread and cereals, milk and milk products, some meat, some fish, margarine, vegetables and fruit.

### The chickens

The RLO feed resulted in a significant increase in EPA, DPA and DHA and a decrease in AA in the chicken breast fillets (Table 4). The chicken is thus a good producer of LC n-3 PUFA from ALA, and chicken meat has potential to be a good source of LC n-3 PUFA in the human diet. The concentration of LC PUFA (AA + EPA + DPA + DHA) made from LA and ALA was 112 mg/100 g in the SO chicken breast muscle and 124 mg/100 g in the RLO group. The percentages of LA + ALA in the two feeds were 40% and 34% of the total fatty acids, respectively, indicating that the synthesis of LC PUFA from LA and ALA was higher in breast muscle from the RLO group compared to the SO group. Thus a diet containing rapeseed and linseed oil appears to trigger the chicken to synthesize LC PUFA. This has also been indicated by others in chickens [4], pigs [19] and bulls [20].

The ability of the chickens and other domestic animals to produce EPA, DPA and DHA from ALA should be valued and given more focus seen in light of the limitations in the world supply of LC n-3 PUFAs from fish and marine sources. To replace the soybean oil (that is now the commonly used feed oil) with linseed and rapeseed oil seems to be an efficient way to increase the intake of LC n-3 PUFA for humans without having to change dietary habits or to take fish oil supplement pills.

As seen from Table 4, the daily intake of EPA + DPA + DHA when eating a portion of 175 g of breast muscle from the RLO chicken would be 142 mg. This is 57% of the proposed EFSA reference intake value of LC n-3 PUFA (250 mg/day) to reduce the risk of CVD [21]. Chicken breast meat from the SO fed group contained 68 mg in 175 g breast meat, thus a portion of the RLO chicken breast meat contained 74 mg more LC n-3 PUFA than the traditional SO chicken. The optimal dose for LC n-3 PUFA remains to be established. The EFSA Panel in 2010 [22] has suggested that 450 mg may be a recommended daily intake of LC n-3 PUFA. This shows that even if all meat consumed had about the same fatty acid composition as the RLO breast meat from this experiment, it would not be enough alone to cover the recommended intake of LC n-3 PUFA.

The chicken thigh meat may be about four to five times higher in fat content compared to the breast meat [4,23], but the percentage of LC n-3 PUFA (g/100 g fatty acids) is lower in thigh meat compared to breast meat [4]. Thus, the LC n-3 PUFA content is somewhat (about 30–50%) higher in thigh muscle compared to breast muscle [4], and by consuming 175 g of the RLO thigh muscle the LC n-3 PUFA intake can be estimated to be about 190 mg instead of 142 mg when consuming the breast muscle.

The concentration of AA was lower in the RLO chicken breast muscle compared to SO. This is in accordance to findings by Poureslami et al. and is shown in both breast muscle and in thigh muscle [4]. The reduction in AA concentration in meat may have implications for the consumer given the nature of the competition between AA and EPA for binding to enzymes and cellular structures [7]. It has been found that purified COX-1 oxygenates EPA at a rate which is only 10% of the rate for AA, while EPA significantly inhibits AA oxygenation by COX-1 [24]. A portion of breast meat from the RLO chicken contained 75 mg AA, while the SO meat contained 128 mg, and the ratio of AA/EPA was only 2 in the RLO chicken breast meat compared to nearly 17 in the meat in the SO group. Such a big difference in AA and EPA balance could be expected to have an impact on the prostanoid synthesis both for the chicken itself and for the consumer eating the chicken.

### The human intervention study

In the present study, an increase in EPA and ALA concentrations, and a decrease in the ratio AA/EPA in serum phospholipids were shown in the persons consuming the RLO chickens. This is in line with the study of Weill et al. and McAfee et al. showing that subjects consuming meat from animals offered a concentrate feed supplemented with linseed oil or a grass based diet had higher LC n-3 PUFA concentrations in erythrocytes, platelets and plasma compared to subjects consuming animal products from animals fed a standard diet [25,26].

The fatty acid composition of serum phospholipids has become established as a valid marker for assessing the status of various fatty acids and to predict dietary fat intakes [27]. As reviewed by Fekete et al. [28], four weeks intervention time and sampling of serum phospholipid fatty acids was a suitable method for studying long-term LC n-3 status in humans.

The content of EPA in 160 g breast muscle from the RLO chicken was 34 mg. In contrast, the content of EPA in the SO chicken breast muscle was only 7 mg per day. Since the participants were not eating oily fish during the study, most of their dietary EPA intake originated from the chicken meat. EPA is synthesized in the body from ALA, but there are variations in the ability to convert ALA to EPA [29-31], and it may be speculated that to some persons a dietary intake of EPA is imperative. The concentration of EPA in serum phospholipids was 1.4 mg/100 ml serum (about 1.3% of FAME) in the RLO treatment group and 0.9 mg/100 ml (about 0.8% of FAME) in the SO group. The EPA concentrations varied between the persons, and at post intervention time three persons in the SO diet group had levels lower than 0.5 mg EPA in phospholipids/100 ml serum (0.5% FAME), but in the RLO group no persons had lower levels than 0.5 mg EPA in phospholipids/100 ml serum post trial.

The sum of EPA plus DHA in serum phospholipids have in populations studies been linked to assess risk of heart disease [32,33]. EPA + DHA levels amounting to more than 4.6% of total fatty acids in serum phospholipids have been associated with a 70% lower risk compared to those with a lower level of these fatty acids [32,33]. In the present study, at baseline, three test subjects in the SO group had less than 4.6% of EPA + DHA (percent of total fatty acids) in serum phospholipids, and five subjects in the RLO were below 4.6%. After the intervention period, two of the three persons in the SO group had reduced their EPA + DHA sum, while all five of the persons in the RLO group improved (increased) their sum of percent EPA + DHA in serum phospholipids. Thus, in the present study, nearly 1/5 of the subjects had less than 4.6% of EPA + DHA in their serum phospholipids. Consumption of the RLO chicken gave an increase in EPA + DHA in serum phospholipids of persons already low in EPA + DHA, and this might theoretically contribute to reduce the risk of coronary heart disease [32,33].

The DPA concentration in serum phospholipids was at about the same level as EPA, but the DHA concentration in serum phospholipids was about five times higher in both treatment groups. In the chicken meat however, it is different; DPA was the most abundant of these three fatty acids, and especially in the RLO chicken there was much DPA (39 mg/100 g meat, Table 4). DPA can be converted to both EPA and DHA [34]. The DPA percentage in serum phospholipids of the subjects eating the RLO chicken showed a tendency to be higher compared to those eating the SO chicken, and to have a significantly lower DPA/EPA ratio (Table 5). No increase in the serum phospholipid concentration of DHA in the test subjects eating RLO chicken meat was observed, although EPA was enhanced. The reasons for this are unknown. One possible explanation might be faster removal of DHA than of EPA from the blood plasma of our test subjects.

The concentration of ALA in serum phospholipids was about 30% higher in the RLO group than the SO group. This is plausible since the amount of ALA in the RLO breast muscle was higher (three times higher) than the SO breast meat.

The ratio AA/EPA was significantly lower in serum phospholipids from the persons eating the RLO chicken. This ratio has been shown to affect the production of different types of eicosanoids and prostanoids [35], and the production of eicosanoids and prostanoids may be altered in a favorable direction towards lower production of thromboxanes of the 2-series which should imply reduced risk of thrombosis.

There were no differences in the concentration of AA in serum phospholipids between the subjects consuming RLO and SO chickens. In the SO group, the AA concentration was higher at post-intervention compared to baseline being 10.2% and 8.4%, (Table 5). It may be speculated that high intake of AA rich meat during the intervention period may increase AA levels in serum phospholipids. However, Kawabata et al. showed no correlations between dietary AA intake and AA in blood lipid fractions [36].

The difference in numbers of men and women in the two intervention groups was of concern, since women have been reported to have a more efficient synthesis of EPA, DPA and DHA from ALA [37]. When calculating the results for men and women separately, there was, however, no difference between the sexes in this study, and the final results would not be different if we excluded the men from the study.

There were no significant differences in serum pre or post trial concentrations of total cholesterol, LDL cholesterol, HDL cholesterol or triacylglycerol in the two intervention groups. This is in accordance to the study of McAfee et al. [26], where the participants were consuming red meat with different amounts of LC PUFAs. Even if the intake of LC n-3 PUFA is nearly twice as high in the RLO group compared to the SO group, the intakes may be too low to significantly affect serum cholesterol or triacylglycerol [38,39].

In the present study with young subjects having normal blood pressure there were no effects on blood pressure when eating the two different meats. Long chain n-3 PUFAs have been shown to have mild antihypertensive effect [11,40], however, the difference in LC n-3 PUFA intake between the two groups may have been too low to reveal any effects. There were no differences in CRP between the two groups. Although research studies have suggested that LC n-3 PUFA may have anti-inflammatory effects [12,39], this was not observed in the present study with young healthy persons.

## Conclusion

Ingestion of chicken meat from chickens fed a diet containing rapeseed plus linseed oil increased EPA and reduced the AA/EPA ratio in serum phospholipids in young healthy persons compared to persons eating chicken meat from birds raised on a diet containing soybean oil (similar to a commercially available chicken). All the five persons who had less than 4.6% of EPA + DHA in their serum phospholipids at baseline, improved (increased) the sum of EPA + DHA after the four week intervention when consuming chickens fed rapeseed plus linseed oil supplementation.

The chicken produces some LC n-3 PUFA from ALA, and chicken meat may become a good dietary source of LC n-3 PUFA provided that the birds are given rapeseed and linseed oil instead of soybean oil. This can be an efficient and easy way to increase the amount of LC n-3 PUFA in the general human diet, without having to make any changes in food habits.

## Abbreviations

AA: Arachidonic acid 20:4n-6; ALA: *Alpha*-linolenic acid 18:3n-3; BMI: Body mass index; CRP: C-reactive protein; DBP: Diastolic Blood Pressure; DHA: Docosahexaenoic acid 22:6n-3; DPA: Docosapentaenoic acid 22:5n-3; EPA: Eicosapentaenoic acid 20:5n-3; HDL: High density lipoprotein cholesterol; LA: Linoleic acid; LC PUFA: Long chain polyunsaturated fatty acids; LDL: Low density lipoprotein cholesterol; PUFA: Polyunsaturated fatty acids; RLO: Rapeseed plus linseed oil; SBP: Systolic Blood Pressure; SO: Soybean oil; TC: Total cholesterol.

## Competing interests

All authors have read and approved the final manuscript. The authors declare no conflict of interest. All authors of this research have no conflict of interest related with employment, consultancies, stock ownership, grants or other funding.

## Authors’ contributions

Each author has participated sufficiently, intellectually or practically, in the work to take public responsibility for the content of the article, including the conception, design, and conduction of the experiment and for data interpretation (authorship). NFN, TJM, MA and AH carried out the study, data analyses, performed the statistical analysis and helped to draft the manuscript. ATH participated in the design, data analyses and writing of the study. All authors read and approved the final manuscript.
